# Supplementary material for: Polish and New Zealand Propolis as Sources of Antioxidant Compounds Inhibit Glioblastoma (T98G, LN-18) Cell Lines and Astrocytoma Cells Derived from Patient
Source: Antioxidants (Basel). 2022 Jun 29;11(7):1305. doi: 10.3390/antiox11071305 (PMC9312157; doi:10.3390/antiox11071305)
Supplement: Supplementary file 1 [file antioxidants-11-01305-s001.zip › Table S1.pdf]

Table S1. Chemical composition of the ethanolic extracts of propolis from Poland (PPE) and New Zealand (MPE).

Components	CAS	I_T^{Exp}	I_T^{Lit}	PPE [%]	MPE [%]
Benzyl alcohol, mono-TMS	14642-79-6	1152	1155	trace*	0.09
2-Phenyl ethanol, mono-TMS	14629-58-4	1225	1227	trace	0.04
Benzoic acid, mono-TMS	2078-12-8	1244	1247	**1.80**	0.33
Diethylene glycol, di-TMS? (73,117,103,147,191)	16654-74-3	1250	1239	-	0.07
H_3PO_4, tri-TMS	10497-05-9	1289	1289	-	0.02
Glycerol, tri-TMS	6787-10-6	1291	1293	0.22	0.57
Succinic acid, di-TMS	40309-57-7	1321	1324	-	trace
Ethyl dihydrocinnamate	2021-28-5	1345	1349	-	trace
Hydroquinone, di-TMS	2117-24-0	1406	1410	0.04	trace
3-Hydroxy acid, di-TMS? (147,73,103,233)	-	1410	-	-	trace
Hydrocinnamic acid, mono-TMS	21273-15-4	1414	1418	trace	0.06
Cinnamoyl alcohol, mono-TMS	N/A**	1425	1427	trace	0.03
3-Hydroxy acid, di-TMS? (73,131,147,233)	-	1461	-	-	0.02
4'-Hydroxyacetophenone, mono-TMS	18803-29-7	1467	1471	-	trace
Malic acid, tri-TMS	38166-11-9	1507	1511	-	0.03
Vanillin, mono-TMS	6689-43-6	1534	1533	trace	trace
Erythritol, tetra-TMS	25258-02-0	1536	1535	-	0.06
Cinnamic acid, mono-TMS	2078-20-8	1542	1546	0.20	**1.82**
7-Phenyl-5-hepten-2-one? (130,129,91)	33046-89-6	1567	-	-	0.03
Protocatechuic aldehyde, di-TMS	N/A	1619	1620	trace	0.07
NN (73>130,75,233,248)	-	1626	-	-	0.15
4-Hydroxybenzoic acid, di-TMS	2078-13-9	1632	1636	0.16	0.03
Docosanoic acid, mono-TMS	55520-95-1	1661	1658	trace	-
Guaiol, mono-TMS	N/A	1682	1685	trace-	0.07
Acorenol, mono-TMS	N/A	1720	1723	-	0.02
Agarospirol, mono-TMS	N/A	1737	1734	-	0.01
γ-Eudesmol, mono-TMS	N/A	1740	1739	-	0.11

α-Bisabolol, mono-TMS	N/A	1747	1752	trace	0.05
β-Eudesmol, mono-TMS	N/A N/A	1749	1751	-	0.09
Arabinitol, penta-TMS	25138-28-7	1755	1760	-	0.07
Benzyl benzoate	120-51-4	1764	1763	0.29	-
Vanillic acid, di-TMS	2078-15-1	1776	1776	trace	-
(Z)-*p*-Coumaric acid, di-TMS	N/A	1799	1798	0.12	-
Methylfuranoside, tetra-TMS	30788-71-7	1811	1813	-	0.11
p-Methoxycinnamic acid, mono-TMS	25436-23-1	1827	1830	-	0.11
Cinnamylideneacetic acid, mono-TMS	N/A	1835	1840	trace	0.63
α-Fructofuranose, penta-TMS	N/A	1841	1845	0.26	0.69
β-Fructofuranose, penta-TMS	N/A	1850	1854	3.67	7.32
α-Mannofuranose, penta-TMS	N/A	1872	1874	-	0.35
α-Glucofuranose, penta-TMS	66807-66-7	1884	1885	-	0.11
NN (73,131,204,368,203)	-	1896	-	-	0.06
α-Glucopyranose, penta-TMS	N/A	1929	1930	0.91	4.02
p-Coumaric acid, di-TMS	10517-30-3	1944	1947	**9.77**	0.87
SesquiterpenolC15H26O-TMS? (131>73…279,103)	-	1948	-	-	0.26
NN (131,73,249,179…399,355)	-	1956	-	-	0.04
Mannitol, hexa-TMS	14317-07-8	1970	1972	-	0.04
Sedoheptulose, hexa-TMS	74987-26-0	1974	1972	-	0.16
Ethyl hexadecanoate	628-97-7	1990	1994	-	0.03
NN (73,147,289,248,319…379)	-	2007	-	-	0.05
β-Glucopyranose, penta-TMS	2775-90-8	2028	2032	0.99	5.25
3,4-Dimethoxycinnamic acid, mono-TMS	27750-71-6	2030	2034	-	1.51
Gluconic acid, hexa-TMS	34290-52-3	2041	2045	-	0.04
Hexadecanoic acid, mono-TMS	55520-89-3	2049	2052	0.27	0.11
(*E*)-1,4-Diphenyl-3-buten-1-one	32363-55-6	2072	-	-	trace
Isoferulic acid, di-TMS	32342-04-4	2087	2088	0.95	0.82
Ethyl caffeate, di-TMS	N/A	2092	2091	-	0.04

NN (131,73>162,143)	-	2096	-	-	0.26
(*E*)-Ferulic acid, di-TM	10517-09-6	2101	2101	**3.22**	0.15
myo-Inositol, hexa-TMS	2582-79-8	2124	2125	trace	0.04
NN (73>157,156)	-	2148	-	-	0.06
3-Methylbutanyl (E)-*p*-coumarate, mono-TMS	N/A	2152	2145	0.65	-
(*E*)-Caffeic acid, di-TMS	10586-03-5	2155	2155	**2.10**	**1.53**
3-Methyl-3-butenyl *p*-coumarate, mono-TMS	N/A	2159	2159	0.18	0.11
NN (247>73,131…358)	-	2169	-	-	0.10
2-Methyl-2-butenyl *p*-coumarate, mono-TMS	N/A	2205	2201	0.97	trace
3-Methyl-2-butenyl *p*-coumarate, mono-TMS	N/A	2212	2216	0.23	0.07
Linoleic acid, mono-TMS	56259-07-5	2217	2215	trace	0.03
Oleic acid, mono-TMS	21556-26-3	2222	2222	0.33	0.21
NN (73>156,244,143,93,147…381)	-	2234	-	1.31	0.17
Octadecanoic acid, mono-TMS	18748-91-9	2249	2252	trace	0.06
3-Methyl-3-butenyl isoferulate, mono-TMS	N/A	2303	2304	-	0.38
3-Methyl-3-butenyl (*E*)-ferulate, mono-TMS	N/A	2318	2319	0.07	0.16
Benzyl (Z)-*p*-coumarate, mono-TMS	N/A	2323	2329	0.07	-
Eicosanoic acid, mono-TMS	55530-70-6	2349	2349	-	0.03
NN (335,73,446,147,69,41,147)	-	2346	-	-	0.04
3-Methylbutanyl (*E*)-caffeate, di-TMS	N/A	2358	2358	0.06	0.15
3-Methyl-2-butenyl (E)-isoferulate, mono-TMS	N/A	2365	2365	-	0.05
3-Methyl-3-butenyl (*E*)-caffeate, di-TMS	N/A	2371	2367	**1.18**	**3.39**
3-Methyl-2-butenyl (*E*)-caffeate, di-TMS	N/A	2374	2375	0.50	0.44
NN (397,369,73,91)	-	2384	-	-	0.03
Pinostrobin chalcone	18956-15-5	2392	-	0.16	0.07
NN (73,75,55,143,207,129,41)	-	2404	-	-	0.02
Cinnamyl cinnamate	122-69-0	2408	2391	-	0.29
2-Methyl-2-butenyl (*E*)-caffeate, di-TMS	N/A	2414	2413	0.09	0.24
2',6'-Dihydroxy-4'-methoxydihydrochalcone,di-TMS	N/A	2418	2416	0.46	0.02

3-Methyl-2-bytenyl (*E*)-caffeate, di-TMS	N/A	2425	2421	**1.65**	**2.36**
NN (143,73,81,95,121,151)	-	2444	-	-	0.02
NN (287,372,357,263,73)	-	2450	-	-	0.18
NN (262,73,247,460,375,287,445)	-	2452	-	-	0.25
Pinocembrin, mono-TMS	N/A	2460	2461	1.14	0.46
2’,6’,α-Trihydroxy-4’-methoxychalcone, tri-TMS	N/A	2491	2492	0.14	-
(*Z*)-Coniferyl benzoate, mono-TMS	N/A	2494	2495	-	0.15
n-Pentacosane	629-99-2	2500	2500	trace	trace
Pinostrobin chalcone, di-TMS	N/A	2506	2508	0.26	0.04
Pinostrobin, mono-TMS	N/A	2512	2512	0.66	0.84
Benzyl (*E*)-*p*-coumarate, mono-TMS	N/A	2516	2515	3.78	0.37
1-*p*-Coumaroyl glycerol, tri-TMS	N/A	2528	2528	0.06	-
Pinocembrin chalcone, tri-TMS	N/A	2542	2541	0.09	0.08
Pinocembrin, di-TMS	N/A	2551	2552	**6.93**	**14.10**
NN (73,75,121,81,95,143…)	-	2555	-	-	0.05
NN (303>73,95,147,213,225)	-	2563	-	-	0.81
NN (262,73,247,460,375)	-	2569	-	-	0.09
2-Acetyl-1-*p*-coumaroyl glycerol, di-TMS	N/A	2578	2578	0.12	-
1-Acetyl-3-*p*-coumaroyl glycerol, di-TMS	N/A	2581	2580	0.19	-
Chalcone, TMS? (192,73,311,238)	N/A	2586	-	trace	0.26
2-Phenylethyl *p*-coumarate, mono-TMS	N/A	2603	2603	1.02	0.11
Pinobanksin, tri-TMS	N/A	2613	2611	**4.25**	**4.73**
3-Hydroxyeicosanoic acid, di-TMS	N/A	2623	2620	-	0.03
Pinobanksin 3-acetate, mono-TMS	N/A	2634	2632	**1.26**	0.21
Coniferyl benzoate, mono-TMS	N/A	2637	2640	trace	-
Chrysin, mono-TMS	N/A	2655	2648	**1.95**	**0.42**
Benzyl (E)-isoferulate, mono-TMS	N/A	2659	2659	-	0.26
2’,6’-Dihydroxy-4,4’-dimethoxydihydrochalcone	N/A	2659	2659	trace	
NN (238,385,325,73,43,341)	-	2666	-	0.34	0.21
Pinobanksin x-acetate, TMS? (296,443,73,383)	-	2671	-	-	0.58

5,7-Dihydroxy-3-methoxyflavanone	N/A	2675	2673	**2.02**	**2.04**
Benzyl (E)-ferulate, mono-TMS	N/A	2680	2680	1.64	0.45
Pinobanksin 3-acetate, di-TMS	N/A	2694	2693	**10.01**	**9.00**
NN (325>>282,155,73)	-	2706	-	0.14	0.85
Sucrose, octa-TMS	19159-25-2	2714	2714	0.25	0.33
Galangin, di-TMS	N/A	2719	2717	trace	trace
Benzyl (E)-caffeate, di-TMS	N/A	2723	2722	3.79	2.70
2’,6’,4-Trihydroxy-4’-methoxydihydrochalcone, tri-TMS	N/A	2636	2637	0.14	-
Pinobanksin 3-propanoate, di-TMS	N/A	2737	-	-	0.06
Isosakuranetin, mono-TMS	N/A	2740	2742	trace	-
Chrysin, di-TMS	N/A	2746	2745	**5.33**	**5.73**
5,7-Dihydroxy-3-methoxyflavone, di-TMS	N/A	2755	2750	0.67	0.60
1-Acetyl-3-caffeoyl glycerol, tri-TMS	N/A	2761	2768	0.06	-
Galangin, tri-TMS	N/A	2767	2769	**8.95**	**9.60**
Disaccharide, TMS	-	2775	-	-	0.12
Pinobanksin 3-isobutanoate, di-TMS	N/A	2788	2791	0.52	0.51
β-Maltose, octa-TMS	N/A	2797	2800	0.07	0.14
CAPE, di-TMS	N/A	2805	2805	**1.29**	**1.15**
Isosakuranetin, di-TMS + disaccharide	-	2816	2820	**1.34**	-
Isosakuranetin, di-TMS	N/A	2816	2820	-	0.18
Dihydroxymethoxyflavone, di-TMS	-	2821	2820	0.39	0.40
Cinnamyl (E)-*p*-coumarate, mono-TMS	N/A	2836	2833	**1.91**	0.23
Tetracosanoic acid, mono-TMS	74367-37-6	2844	2845	0.53	-
Pinobanksin-3-*n*-butanoate, di-TMS	N/A	2848	2849	0.17	0.13
Disaccharide, TMS (73,361,217)	-	2857	-	-	0.09
Sakuranetin chalcone, tri-TMS	N/A	2871	2871	-	0.05
Sakuranetin , di-TMS	N/A	2877	2880	0.55	0.05
Pinobanksin 5-pentanoate, di-TMS	N/A	2885	2884	0.19	0.58
β-Cellobiose, octa-TMS	N/A	2889	2888	-	0.12

Naringenin, tri-TMS	N/A	2895	2895	0.23	0.06
NN (191,117,91)	-	2933	-	-	0.03
Disaccharide, TMS (204,73,361)	-	2956	-	-	0.03
Pinobanksin 5-pentenoate, di-TMS	N/A	2965	2964	-	0.03
NN (73,299,305,147,129,233…445)	-	2968	-	-	0.05
Cinnamyl (E)-isoferulate, mono-TMS	N/A	2980	2975	-	0.56
Cinnamyl (E)-ferulate, mono-TMS	N/A	2990	2997	0.09	-
NN (356,341,75,135)	-	2995	-	0.26	-
β-Isomaltose, octa-TMS	N/A	3005	3005	-	0.05
3,5,7-Trihydroxy-4’-mathoxyflavone, tri-TMS	N/A	3015	3015	0.31	-
Pinobanksin 3-hexanoate, di-TMS	N/A	3037	3032	-	0.04
Cinnamyl (E)-caffeate, di-TMS	N/A	3044	3043	0.41	0.96
Kaempheride, tri-TMS	N/A	3052	3050	0.33	0.02
9-Hentriacontene	-	3076	3075	0.14	-
Kaempherol, tri-TMS	N/A	3082	3078	0.29	0.04
NN (414,399)	-	3086	-	0.41	0.03
NN (444,401,73,429)	-	3096	-	0.18	0.02
3',4',7-Trihydroxyisoflavone, tri-TMS	N/A	3101	3098	0.14	0.09
Kaempherol, tetra-TMS	N/A	3114	3114	0.38	0.41
NN (341,73,103,143…475,515)	-	3121	-	-	0.05
5,7,4'-Trimethyl-3-methoxyflavone, tri-TMS	N/A	3141	3139	-	0.09
Apigenin, tri-TMS	N/A	3163	3161	-	0.09
Triterpenoid (189,73,129,143,305)	-	3180	-	-	0.08
Quercetine, penta-TMS	4067-66-7	3218	3213	0.11	-
Isorhamnetin, tetra-TMS	N/A	3245	3245	-	0.22
p-Coumatate or ferulate, TMS (219,205,249)	N/A	3249	-	0.21	-
NN (73,271,301,103,129,147…451,531)	-	3259	-	-	0.11
7-Tritriacontene	N/A	3283	3282	0.22	-
Myricetin, hexa-TMS	N/A	3303	3303	-	0.04
Triterpenoid, TMS (73,189,271,375,129,143)	-	3311	-	0.28	0.14

NN (73,301,299,461)	-	3436	-	-	0.11
Triterpenoid, TMS (189,73)	-	3497	-	-	0.11
NN (393,207,73,134,129)	-	3574	-	-	0.22
1,3-Di-*p*-coumaroyl glycerol, tri-TMS	N/A	3869	3869	0.02	-
2-Acetyl-1,3-di-*p*-coumaroyl glycerol, di-RMS	N/A	3963	3963	0.89	-
Total				100.00	100.00

* trace – below 0.01% of the total ion current. ** N/A - not available
